# ALKBH1 knockdown promotes the growth, migration and invasion of HTR-8/SVneo cells through regulating the m5C modification PSMD14

**DOI:** 10.1038/s41598-025-91233-3

**Published:** 2025-03-01

**Authors:** Caili Zhang, Jie Li, Luwen Wang, Peifeng Yang, Xiaohua Luo

**Affiliations:** Department of Obstetrics and Gynecology, The Third Affiliated Hospital of Zhengzhou University or Maternal and Child Hospital of Henan Province, No.10, Kangfuqian Street, Zhengzhou City, 450001 Henan China

**Keywords:** Preeclampsia, ALKBH1, 5-methylcytosine methylation modification, PSMD14, Cell biology, Pathogenesis

## Abstract

Preeclampsia (PE) is a pregnancy disease characterized by insufficient invasion and growth of trophoblast cells. adeno-associated virus encoding alkB homolog 1 (ALKBH1) is a demethylase in 5-methylcytosine (m5C) methylation modification. This study was performed to explore the role of ALKBH1 in hypoxia treated human extravasated trophoblast cells. Hypoxia treated human extravasated trophoblast cells (HTR-8/SVneo) was used to simulate the occurrence of PE in vitro. The cells phenotype was detected by CCK-8 and Transwell assays. The m5c levels and m5C levels of PSMD14 were analyzed by m5C dot blot and M5C Me-RIP assays. Then, the interaction between ALKBH1 and PSMD14 were confirmed by RIP and dual-luciferase reporter assays. ALKBH1 was up-regulated in hypoxia treated HTR-8/SVneo cells. Additionally, ALKBH1 knockdown increased the m5C contents, cell viability, migration and invasion abilities of hypoxia treated HTR-8/SVneo cells. Furthermore, ALKBH1 knockdown increased the m5C and mRNA levels, and mRNA stability of PSMD14. RIP and dual-luciferase reporter assays demonstrated that ALKBH1 interacted with PSMD14. Besides, PSMD14 knockdown reversed the effects of ALKBH1 silencing on cell viability, migration and invasion abilities of hypoxia treated HTR-8/SVneo cells. ALKBH1 mediated m5C levels were decreased in the hypoxia treated HTR-8/SVneo cells, which further decreased the cell viability, migration and invasion abilities through targeting the PSMD14 levels.

**Supplementary Information:**

The online version contains supplementary material available at 10.1038/s41598-025-91233-3.

## Introduction

Preeclampsia (PE) is a pregnancy disease characterized by hypertension and proteinuria in pregnant women after 20 weeks of pregnancy^1^. The incidence rate of PE in China is about 9.4%~10.4%, which can increase the risk of adverse pregnancy outcomes for pregnant women and newborns, and even lead to maternal and newborn deaths^2^. At present, research on the pathogenesis of PE mainly focuses on placental factors, genetic factors, immune factors, etc., but these multiple theories have not fully explained the specific causes of PE^3^;^4^. Insufficient invasion and growth of trophoblast cells affect placental implantation and hypoperfusion, which may be important reasons for a series of clinical symptoms in PE pregnant women^5–7^. Therefore, exploring the molecular regulatory mechanisms that affect the proliferation and invasion of trophoblast cells is of positive significance for targeted molecular therapy of preeclampsia.

5-methylcytosine (m5C) methylation modification refers to a highly concentrated and reversible epigenetic modification that connects a methyl group on the 5th carbon of the cytosine ring in DNA or RNA^8^. The dynamic m5C methylation and demethylation modifications are mainly mediated by three proteins, namely methyltransferase (writer), demethylase (eraser), and m5C recognition protein (reader)^9^. In recent years, multiple studies have found that m5C modification participates in the regulation of multiple diseases by regulating the RNA stability of target genes^10^;^11^. However, researches on m5C modification is mostly focused on tumors^12–14^, and there are few reports on pregnancy related diseases such as PE. Recently, a new study found that the total peak of m5C methylation in placental tissue of PE patients is reduced, and differentially expressed m5C modifying genes are enriched in hypoxia process^15^. This indicates that changes in m5C modification may play a certain role in the pathogenesis of PE. In m5C modification progress, adeno-associated virus encoding alkB homolog 1 (ALKBH1), as a key gene for demethylation modification^16^, has not been reported in PE research. Given the discovery of low m5C modification levels in PE patients, we speculate ALKBH1 mediated de m5C modification may be a potential factor.

Proteasome 26 S subunit, non-ATPase 14 (PSMD14), also known as RPN11 or POH1, is a non ATPase regulatory subunit in the 26 S proteasome. PSMD14 belongs to the JAMMs isopeptidase family and contains the JAMM domain, which can only exert its deubiquitinase activity under Zn^2+^dependent conditions^17^. PSMD14 can remove ubiquitin chains from target proteins to promote further degradation of substrates in proteasomes, then regulating the growth and development of tumor cells^18^;^19^. Recently, a study found that the expression of PSMD14 is reduced in PE patients, and upregulation of PSMD14 expression promoted the proliferation and invasion of placental trophoblast cells^20^. However, it is still unknown whether the expression of PSMD14 is regulated by ALKBH1 mediated m5C modification in PE.

Therefore, this study aimed to simulate the occurrence of PE in vitro by using hypoxia treated human extravasated trophoblast cells (HTR-8/SVneo). Thus exploring the regulatory effects of ALKBH1 mediated m5C modification of PSMD14 on cell growth, migration, and invasion abilities of HTR-8/SVneo cells.

## Materials and methods

### Cell culture and treatment

Human extravasated trophoblast cells (HTR-8/SVneo) were purchased from Pricella (Wuhan, China) and cultured with RPMI-1640 medium (Pricella) containing 10% fetal bovine serum (FBS, Sigma, USA) and 1% penicillin/streptomycin (Sigma). The cultivation condition is set to 37 ℃ and 5% CO_2_. For PE model establishment, HTR-8/SVneo cells were cultured in an AW200SG hypoxic workstation (Electrotek, UK), the cultivation condition is set to 1% O_2_, 5% CO_2_, and 94% N_2_ at the temperature is set to 37 ℃. The cells in the control group were cultured at 20% O_2_.

### Cell transfection

Short hairpin RNA ALKBH1 (shALKBH1), short hairpin RNA PSMD14 (shPSMD14), and shRNA negative control (shNC) were synthesized and provided by Thermo Fisher Scientific (MA, USA). HTR-8/SVneo cells (5 × 10^5^ cells/well) were cultured in 6-well plates and transfected with shALKBH1, shPSMD14, or shNC using Lipofectamine 3000 (Thermo Fisher) for 48 h. The knockdown efficiency of shALKBH1 or shPSMD14 was tested by RT-qPCR.

### Real-time quantitative polymerase chain reaction (RT-qPCR)

Total RNA was extracted from cells using TRIzol reagent (Invitrogen). RNA concentration was determined using the NanoDrop™ One/OneC ultramicro-ultraviolet spectrophotometer (ThermoFisher Scientific). Subsequently, BiomarkerScript III RT Master Mix for qPCR kit was used to reverse transcribed RNA into cDNA (Biomarker Technologies Co. LTD., Beijing, China), and the RT-qPCR amplification experiment was performed using the Biomarker 2 × SYBR Green Fast qPCR Mix (Biomarker) with the reaction conditions: 95°C for 3 min, 40 cycles of 95°C for 5 sec, 60°C for 30 sec, and a melt curve stage. Finally, the relative RNA expression was calculated using the 2^−ΔΔCt^ method. GAPDH served as the normalization. Main primer sequences (5’ -> 3’)used in this study were listed as follows:

ALKBH1, Forward Primer AAACTTTTCCGCTTCTACCGTC, Reverse Primer TTTGAGTCCATAGGCTTGCCA;

PSMD14, Forward Primer AAGTTATGGGTTTGATGCTTGGA, Reverse Primer ATACCAACCAACAACCATCTCC;

GAPDH, Forward Primer GGAGCGAGATCCCTCCAAAAT, Reverse Primer GGCTGTTGTCATACTTCTCATGG.

For mRNA stability of PSMD14 determination, HTR-8/SVneo cells were treated with 5 µg/mL actinomycin D (Sigma) for 0, 4, 8, and 12 h. At each pointed time, PSMD14 expression was measured using qRT-PCR after ALKBH1 knockdown.

### Cell counting kit-8 (CCK-8) assay

CCK-8 kit (Beyotime, Shanghai, China) was used to analyze cell viability. The cells were seeded into a 96-well plate at the density of 1 × 10^3^ cells/well, and cultured fie 24 h. Three replicate wells were set up. Then 10 µL of CCK-8 solution was added to each well to incubate with cells for 2 h. Finally, a microplate reader (Thermo Fisher) was used to assess the absorbance at 450 nm.

### Western blot

The protein of cells was obtained using the RIPA cell lysate (Beyotime). Then the protein sample was heated in boiling water bath for 10 min to obtain the loading amount of 40 mg. The membrane was separated by SDS-PAGE and transferred to PVDF membrane. The membrane was cut according to the molecular weight of the target protein. The membrane was enclosed in 5% skim milk at room temperature for 2 h. Primary antibodies ALKBH1 (ab128895, 1/1000, Abcam), PSMD14 (ab109123, 1/1500, Abcam and GAPDH (ab9485, 1/2500, Abcam) were added, incubated overnight at 4℃. After washed with with TBST for 4 times, 10 min each time, the IgG H&L (Alexa Fluor^®^ 790) (ab175781, 1/10000, Abcam) secondary antibody was added to the membrane. Finally, protein bands were visualized using the Enhanced Chemiluminescence (ECL) detection system (Thermo Fisher) and imaged with a ChemiDoc MP Imaging System (Bio-Rad Laboratories).

### Transwell assay

The migration and invasion abilities of cells were detected by Transwell assay. In brief, transwell chambers (8 μm pore size, 24-well) without Matrigel were used for cell migration determination, and Matrigel-coated chambers were used for cell invasion determination. HTR-8/SVneo cell suspension was added in the upper chambers, and the complete medium was added to fill the bottom chambers. After 24 h, the cells through the pore were fixed with 4% paraformaldehyde and stained with 0.1% crystal violet. The stained cells were observed using a light microscope (Olympus, Japan).

### M5C Dot blot assay

Total RNA obtained in RT-qPCR was treated 10 mM Tris-EDTA buffer. Then the RNA samples were transferred into Hybond-N + membranes (Beyotime, China). The After drying, the membrane was crosslinked for 1 min (254 nm UV) and blocked with 5% milk for 90 min. After that, the membrane was incubated with an m5C antibody (Abcam, USA) at 4 ℃ for 12 h. Next, the membrane was washed with TBST for three times and then incubated with secondary antibody (HRP-conjugated anti-rabbit IgG, Abcam, USA) for 90 min. Finally, the membrane was visualized using an enhanced chemiluminescence kit (Beyotime).

### M5C methylated RNA Immunoprecipitation assay

M5C expression of PSMD14 was measured by a GenSeq^®^ m5C MeRIP kit (Cloudseq, Shanghai, China). In brief, total RNA obtained in RT-qPCR was fragmented using a PCR system at 70 °C. PGM beads were incubated with 2 µL of m5C antibody or anti-IgG at room temperature for 1 h. Fragmented RNA was incubated with pre-treated beads, and IP buffer at 4 °C for a night. After washing, RNA was purified and PSMD14 expression was measured using qRT-PCR.

### Dual-luciferase reporter assay

The m5C modification sites in PSMD14 were predicted using Rnanut online database (http://www.rnanut.net/rnam5cfinder/). The top three probability scores sites were selected, sites 1# (site147), 2# (site 370), and 3# (site 1326). Then the cDNA containing full-length 3’-UTR of PSMD14 was cloned into the pGL3 luciferase reporter vector (Promega, Madison, WI, USA) to obtain pGL3-PSMD14-wild type (WT). Besides, the sites with the top three probability scores were chosen, and named sites 1# (site147), 2# (site 370), and 3# (site 1326). The pGL3-PSMD14-mutant type (MUT 1#, MUT 2#, MUT 3#) was obtained by the introduction of mutations into pGL3-PSMD14-WT using the QuikChange II Site-Directed Mutagenesis kit (Agilent Technologies, Santa Clara, CA, USA). Next, shALKBH1 and shNC were co-transfected into cells using Lipofectamine 3000 for 48 h. Afterwards, luciferase activity was measured using Dual-Luciferase^®^ Reporter Assay System Kit (Promega) and normalized to the activity of Renilla luciferase. The ratio of firefly/Renilla luciferase activity was used as the relative luciferase activity.

### RNA binding protein Immunoprecipitation (RIP)

The interaction between ALKBH1 and PSMD14 mRNA was evaluated using a RIP kit (Millipore, USA). In brief, HTR-8/SVneo cells (2 × 10^7^) were washed with PBS and lysed using 400 µL complete RIP lysis buffer for 30 min on ice. The the cells were centrifuged at 12,000 rpm for 10 min. After that, the obtained supernatant was divided into input (100 µL), IgG (150 µL), and IP/ALKBH1 (150 µL) groups. Protein A/G magnetic beads were incubated with 5 µg anti-IgG or anti-ALKBH1 at room temperature for 1 h. The supernatant was incubated with antibody-beads complex and RIP buffer at 4 °C overnight. After washing, RNA was purified and PSMD14 expression was measured using qRT-PCR.

### Statistical analysis

All data was analyzed using the SPSS 26.0 software. Student’s t-test or one-way ANOVA followed by Tukey’s post hoc test were used to analyze the differences between two groups or among multiple groups. Data were represented as mean ± standard deviation. *P* < 0.05 was considered statistically significant.

## Results

### ALKBH1 was upregulated in the hypoxia treated HTR-8/SVneo cells

**Fig. 1:**
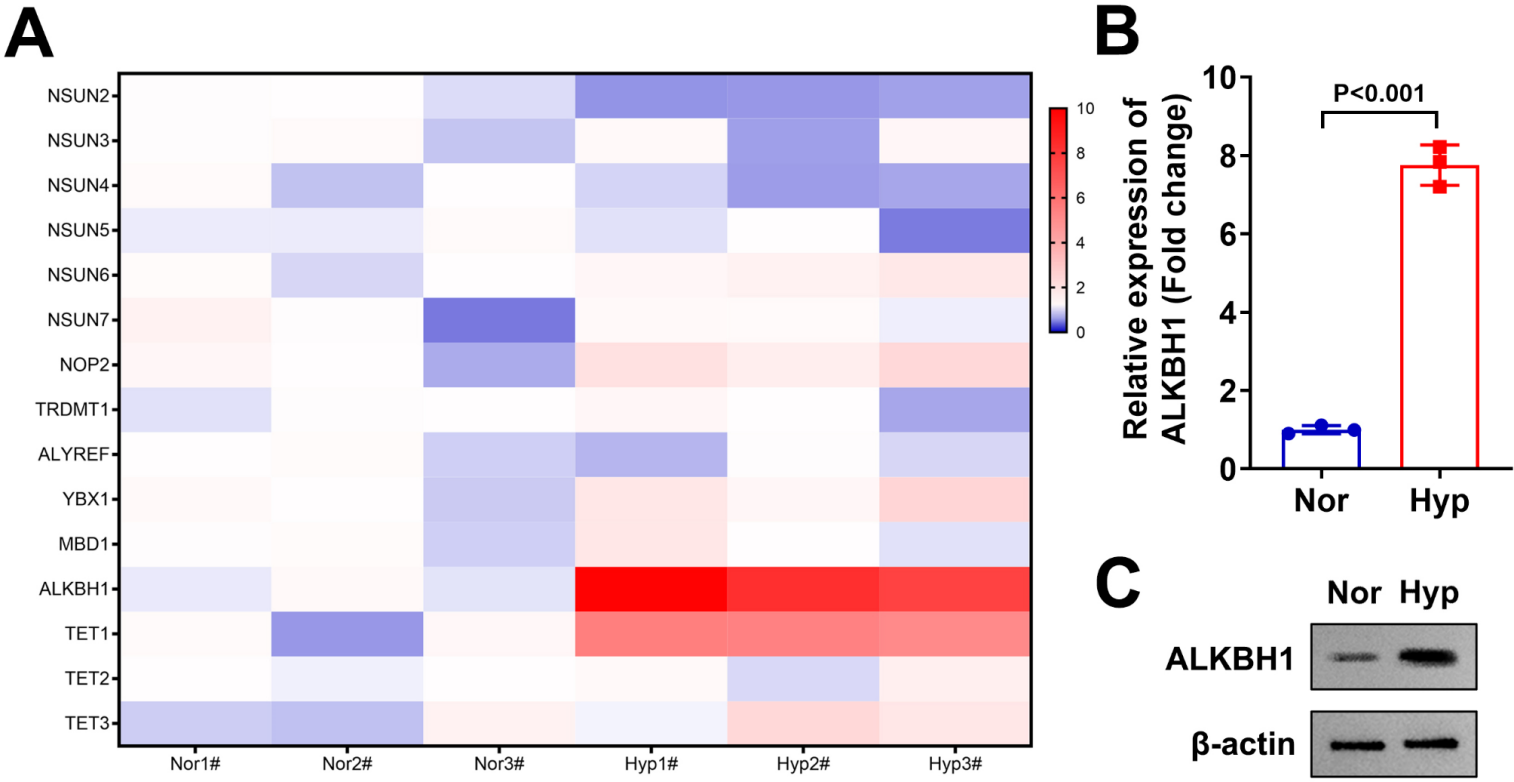
ALKBH1 was upregulated in the hypoxia treated HTR-8/SVneo cells. (**A**) The mRNA levels of m5C RNA modification related genes in the hypoxia treated HTR-8/SVneo cells were detected by RT-qPCR assay. The results were expressed as hot map. (**B**) The quantization of mRNA levels of ALKBH1 in the hypoxia treated HTR-8/SVneo cells. (**C**) The protein levels of ALKBH1 in the hypoxia treated HTR-8/SVneo cells were detected by western blot.

Previous study has demonstrated that m5C RNA modification was closely related to PE progression^15^. Here, the HTR-8/SVneo cells were treated with hypoxia to establish the PE model in vitro^21^ to analyze the expressions of m5C RNA modification related genes. The results were expressed as hot map (Fig. 1A). We found that after hypoxia treatment, NUSN2 levels were significantly decreased (blue colour), ALKBH1 and TET1 were significantly increased (red colour). Among them, the expression change in ALKBH1 was most significant, which was selected for next experiments. Then the mRNA (Fig. 1B) and protein (Fig. 1C) expression of ALKBH1 was increased in the hypoxia treated HTR-8/SVneo cells.

### ALKBH1 knockdown promoted the growth, migration and invasion of hypoxia treated HTR-8/SVneo cells

**Fig. 2:**
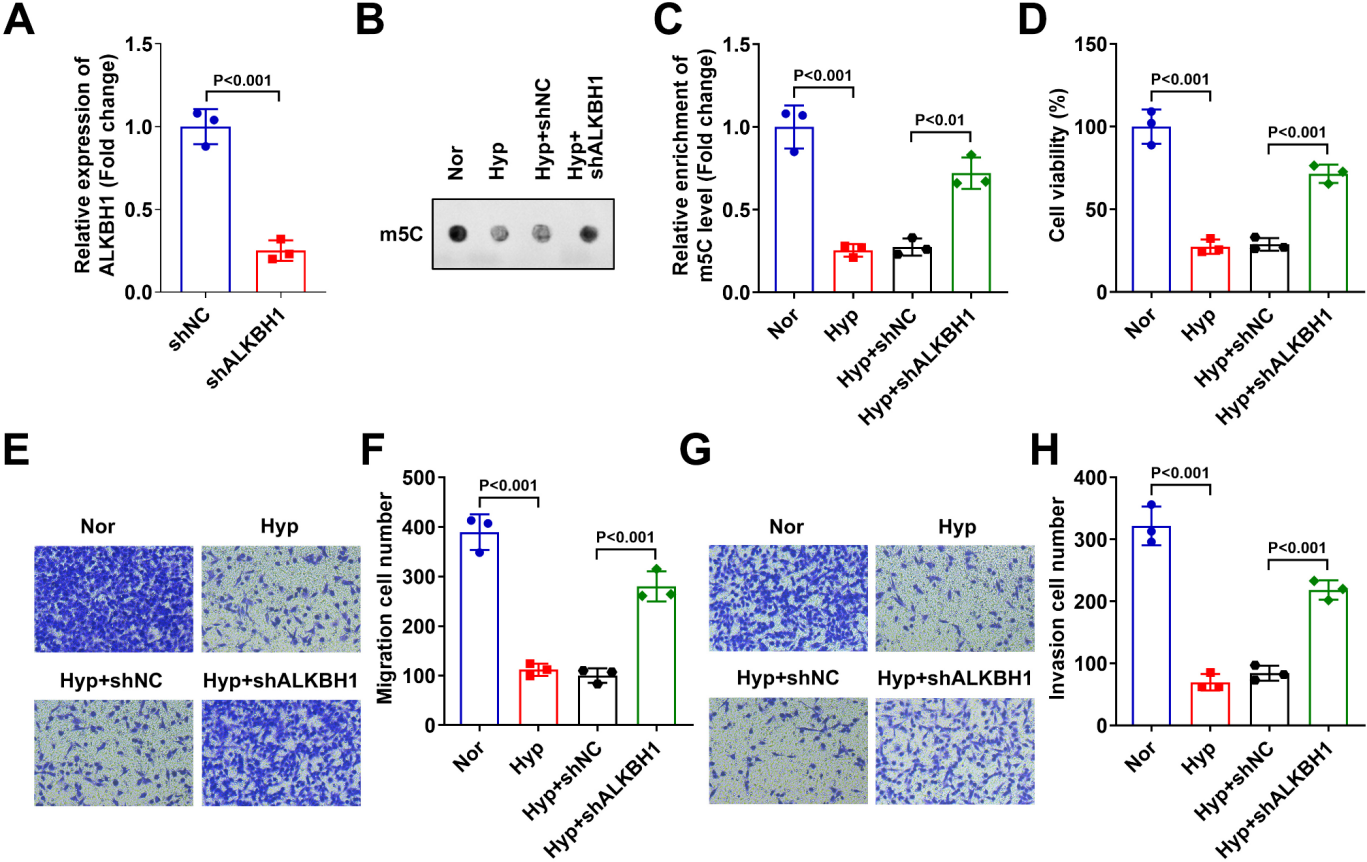
ALKBH1 knockdown promoted the growth, migration and invasion of hypoxia treated HTR-8/SVneo cells. (**A**) The knockdown efficiency of shALKBH1 was validated through RT-qPCR. After hypoxia treatment and shALKBH1 transfection, (**B** & **C**) the m5C levels of HTR-8/SVneo cell were measured by m5C dot blot assay. (**D**) The cell viability of HTR-8/SVneo cells were detected by CCK-8 assay. The migration (**E** & **F**) and invasion (**G** & **H**) abilities of HTR-8/SVneo cells were detected by transwell assay.

Then, the ALKBH1 levels were silenced in hypoxia treated HTR-8/SVneo cells after shALKBH1 transfection (Fig. 2A). The m5C levels (Fig. 2B & C), cell viability (Fig. 2D), migration (Fig. 2E & F) and invasion (Fig. 2G & F) abilities were significantly decreased in the HTR-8/SVneo cells after hypoxia treatment. After ALKBH1 knockdown, the m5C levels (Fig. 2B & C), cell viability (Fig. 2D), migration (Fig. 2E & F) and invasion (Fig. 2G & F) abilities were significantly increased in the hypoxia treated HTR-8/SVneo.

### ALKBH5 interacted with PSMD14 through m5C modification

**Fig. 3:**
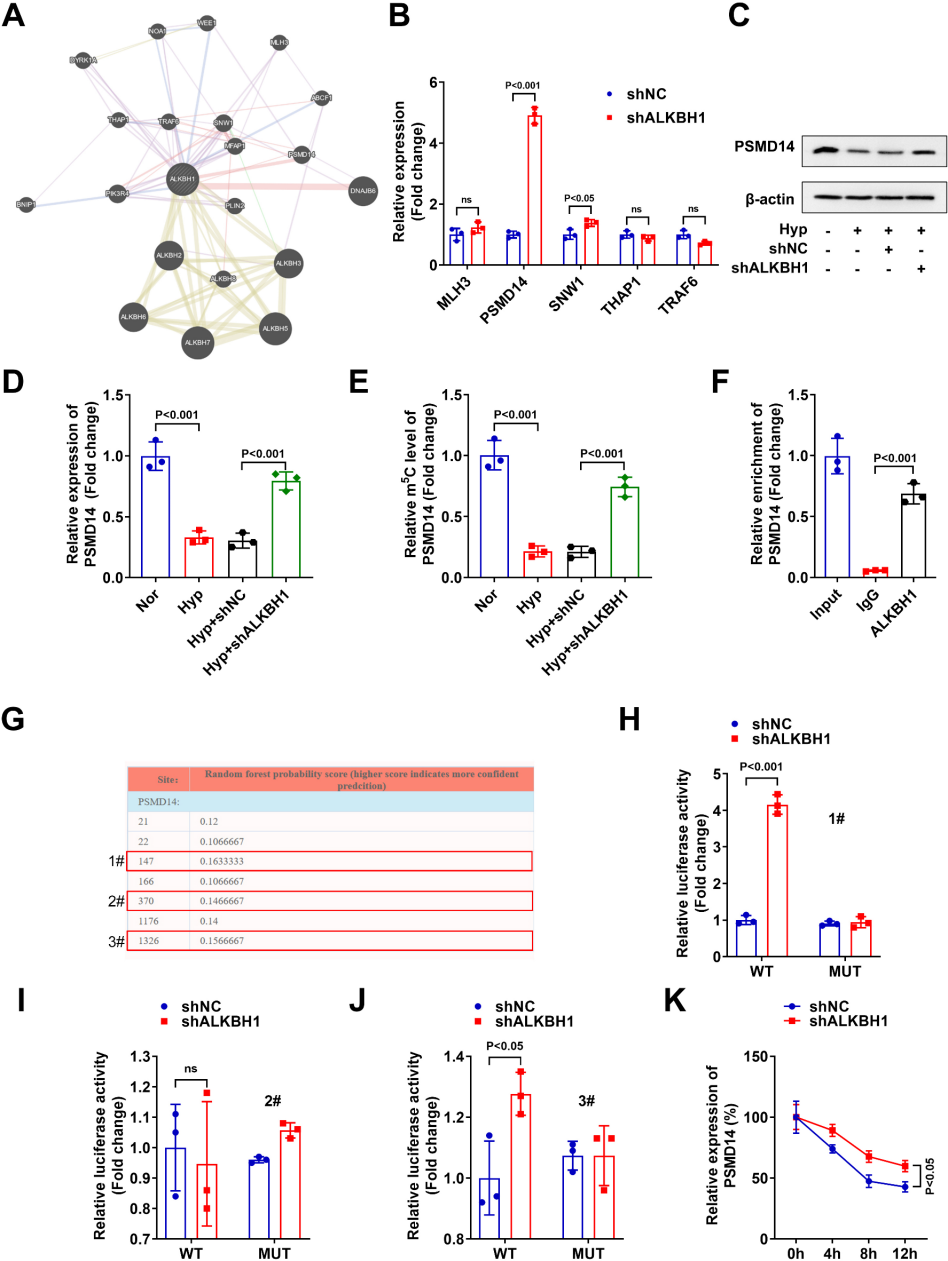
ALKBH5 interacted with PSMD14 through m5C modification. (**A**) PPI network diagram of ALKBH5 related genes. (**B**) The levels of MLH3, PSMD14, SNW1, THAP1 and TRAF6 were detected by RT-qPCR after ALKBH5 knockdown. The protein (**C**) and mRNA (**D**) levels of PSMD14 were detected by western blot and RT-qPCR in the hypoxia treated HTR-8/SVneo cells after ALKBH5 knockdown. (**E**) The m5C levels of PSMD14 were detected by m5C MeRIP kit in the hypoxia treated HTR-8/SVneo cells after ALKBH5 knockdown. (**F**) The interaction between ALKBH1 and PSMD14 was tested by RIP assay. (**G**) The m5C modification sites of PSMD14 were predicted by Rnanut online database. (**H-J**) Luciferase reporter assay was performed to confirm the relationship between ALKBH1 and PSMD14 after mutation of m5C modification sites in PSMD14. (**K**) The mRNA stability of PSMD14 was tested bu RT-qPCR after ALKBH1 knockdown.

Subsequently, through the PPI network diagram (https://string-db.org/), we found ALKBH5 was related to many genes (Fig. 3A). The directly related genes included MLH3, PSMD14, SNW1, THAP1 and TRAF6, and after ALKBH5 knockdown, just PSMD14 and SNW1 levels were significantly increased (Fig. 3B). Additionally, the expression change in PSMD14 was most significant, which was selected for next experiments. The protein (Fig. 3C) and mRNA (Fig. 3D) levels of PSMD14 were significantly decreased in the hypoxia treated HTR-8/SVneo cells, and increased after ALKBH5 knockdown. In the hypoxia treated HTR-8/SVneo cells, the m5C levels of PSMD14 were significnatly decreased. After ALKBH5 knockdown, the m5C levels of PSMD14 were significantly decreased (Fig. 3E). The RIP assay showed that ALKBH1 significantly increased the relative enrichment of PSMD14 compared to IgG, which indicated the interaction between ALKBH1 and PSMD14 (Fig. 3F). Through the Rnanut online database, we found that there were many m5C modification sites in PSMD14 (Fig. 3G). The 147, 370 and 1326 sites (with high score) of PSMD14 were selected to construct mutated genes for luciferase reporter assay. The results showed that in site 1# and site 2# of PSMD14 gene, ALKBH1 knockdown significantly increased the relative luciferase activities, and the luciferase activity change in site 1# was most significant. After site 1# and site 2# mutation, ALKBH1 knockdown showed no influence on the relative luciferase activities. Besides, ALKBH1 knockdown showed no influence on site 2# of PSMD14 (Fig. 3H-J). Finally, after ALKBH1 knockdown, the mRNA stability of PSMD14 was significantly decreased (Fig. 3K). These results indicated that ALKBH1 regulated the PSMD14 levels through modulating the m5C levels on site 1# of PSMD14.

### PSMD14 knockdown reversed the role of ALKBH1 Silencing hypoxia treated HTR-8/SVneo cells

**Fig. 4:**
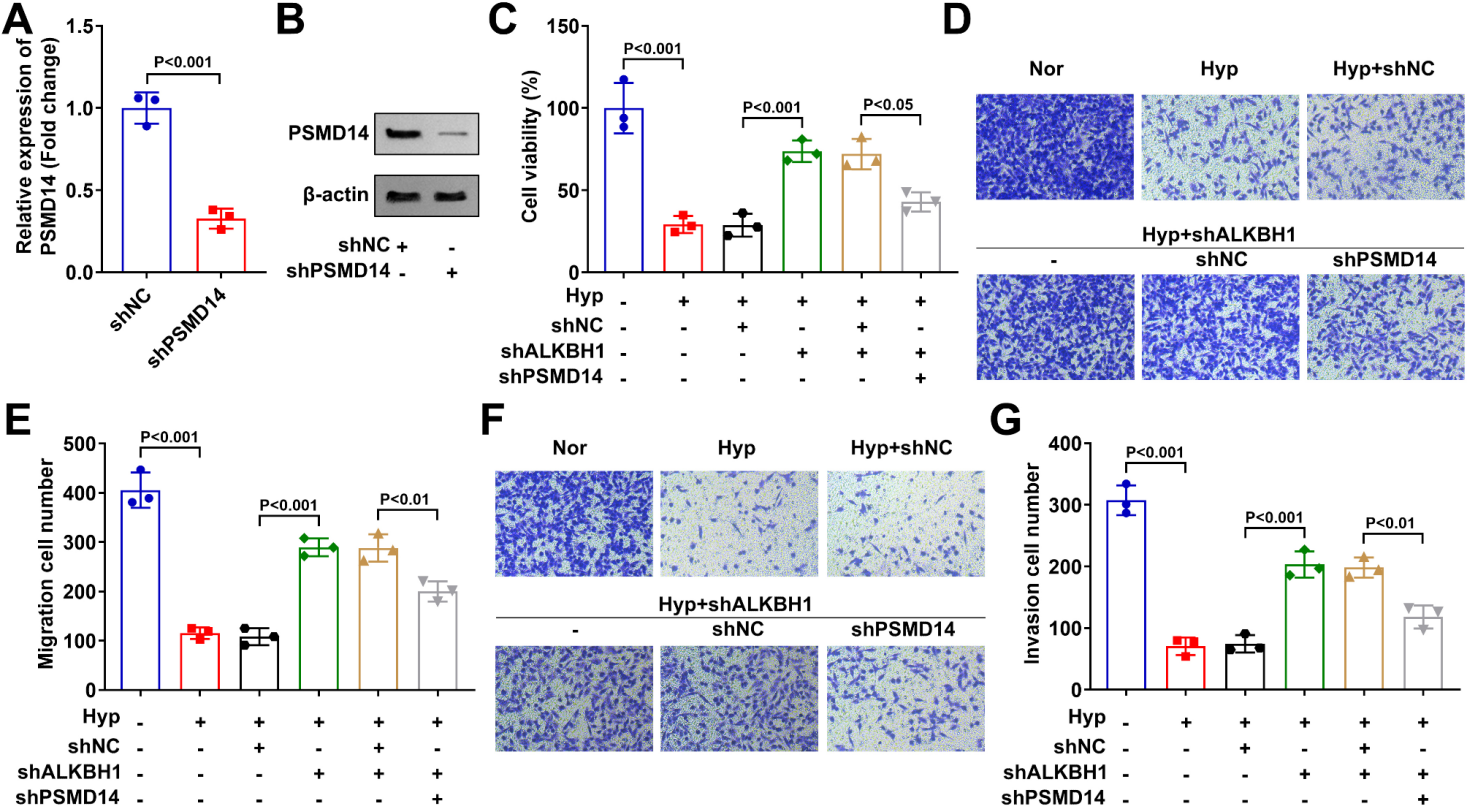
PSMD14 knockdown reversed the role of ALKBH1 silencing hypoxia treated HTR-8/SVneo cells. (**A** & **B**) The knockdown efficiency of shPSMD14 was validated through RT-qPCR and western blot. After hypoxia treatment, shALKBH1 and shPSMD14 transfection, (**C**) the cell viability of HTR-8/SVneo cells were detected by CCK-8 assay. The migration (**D** & **E**) and invasion (**F** & **G**) abilities of HTR-8/SVneo cells were detected by transwell assay.

Finally, in the hypoxia treated HTR-8/SVneo cells, we simultaneously knock out ALKBH1 and PSMD14 to verify whether ALKBH1 participates in the regulation of cell phenotype by regulating the expression of PSMD14. ShPSMD14 transfection significantly decreased the mRNA (Fig. 4A) and protein (Fig. 4B) levels of PSMD14 in the HTR-8/SVneo cells. Then, after PSMD14 silencing, the cell viability (Fig. 4C), migration (Fig. 4D & E) and invasion (Fig. 4F & G) abilities were significantly decreased in the hypoxia treated and shALKBH1 transfected HTR-8/SVneo cells. These results indicated that PSMD14 knockdown reversed the role of ALKBH1 silencing in hypoxia treated HTR-8/SVneo cells.

## Discussion

Trophoblast cells are key factors in the normal growth and development of the fetus, and their functional abnormalities are closely related to the pathogenesis of PE^22^. According to the “two-stage” theory of PE pathogenesis, the first stage is the reconstruction disorder of uterine spiral arteries, resulting in placental ischemia and hypoxia, which leads to apoptosis of trophoblast cells and abnormal release of various placental factors; the second stage is the abnormal release of placental factors into maternal blood circulation, causing endothelial damage and PE-related clinical manifestations^23^. Previous studies have found that the growth, migration, and invasion of placental trophoblast cells are severely damaged by hypoxia treatment. Feng et al.^24^ found under hypoxia, the migration and invasion abilities of HTR-8/SVneo cell was significantly decreased, which was also confirmed by Li et al.^25^. Here, we also demonstrated that hypoxia treatment inhibited the cell viability, migration and invasion abilities of HTR-8/SVneo cells. Hence one can see that hypoxia induced insufficient invasion and growth of trophoblast cells might be a key factor for PE progression.

Recently, abnormal m5C modification has been demonstrated to regulate the cell growth and invasion in many diseases^10^;^26^. In cancer progression, high levels of m5C modification promoted the cell growth, migration and invasion. For example, Hu et al.^27^ demonstrated that NSUN2 mediated m5C modification promoted the proliferation, migration, and invasion of gastric cancer cells. Similarly, Zhang et al.^28^ confirmed that NSUN2 mediated m5C modification was increased in pancreatic cancer tissues and related to aggressive clinical features. NSUN2 silencing weakened the capability of proliferation, migration and invasion of pancreatic cancer cells. Currently, researches on m5C modification are mostly focused on tumors. It is still unknown whether its pro-proliferative effect on tumor cells also occurs in other diseases. Recently, Wei et al.^15^ found that m5C mRNA methylation were decreased in placental tissues from PE patients, and confirmed a potential relationship between m5C levels and the pathogenic mechanisms of PE. Here, we further explored the expressions of m5C modification related genes in hypoxia treated HTR-8/SVneo cells, which showed ALKBH1, a demethylase, was significantly increased. After ALKBH1 knockdown, the insufficient invasion and growth induced by hypoxia was relieved. At same time, the m5C levels in hypoxia treated HTR-8/SVneo cells were also enhanced. These result further explained the role of m5C mRNA methylation in PE was mediated by ALKBH1.

ALKBH1, a member of the AlkB family of Fe(II)/α-ketoglutarate-dependent dioxygenases, has been identified as an enzyme capable of oxidatively demethylating methylated bases in both single-stranded and double-stranded DNA^29^ as well as RNA^30^. In the context of this study on PE, we have observed that ALKBH1 plays a critical role in regulating m5C modifications specifically in PSMD14 within hypoxia-treated HTR-8/SVneo cells, thereby affecting cell viability, migration, and invasion. Beyond its involvement in PE, ALKBH1’s function extends to various biological processes. It contributes to the maintenance of genomic integrity by repairing alkylation damage in DNA^31^. Moreover, ALKBH1 has also been implicated in the regulation of gene expression at the post-transcriptional level through its activity on RNA modifications. The ability of ALKBH1 to demethylate N1-methyladenosine (m1A)^32^ and 3-methylthymine (m3T)^33^ in RNA suggests its importance in controlling RNA stability, splicing, export, and translation. Therefore, ALKBH1 not only influences cellular behavior in the setting of trophoblast cell function but also participates widely in epigenetic regulation, with implications for development, differentiation, and disease states. Thus, the dual functionality of ALKBH1 in both DNA and RNA demethylation underscores its versatile role in cellular homeostasis and highlights potential therapeutic targets for diseases where methylation patterns are aberrant.

Subsequently, in order to investigate which gene ALKBH1 participates in regulating cell phenotype through targeting, we conducted PPI network analysis and found that PSMD14 is one of the key genes regulated by ALKBH1. PSMD14 has been recently demonstrated to decrease in placenta of pregnant women with PE^34^. As reported by Zhang et al.^20^, PSMD14 enhanced the proliferation as well as invasion of trophoblast, which might be a potential targets for PE treatment. In cancer progression, high levels of PSMD14 also enhanced the proliferation, migration and invasion cancer cells, such as breast cancer^18^, ovarian cancer^19^, bladder cancer^35^, etc. It can be seen that increasing the expression of PSMD14 and thus enhancing its promotion of cell growth and invasion may be an effective means of promoting the growth of placental trophoblast cells. Here, we found that ALKBH1 knockdown enhanced the m5C levels and mRNA expressions of PSMD14, which further demonstrated by increase of mRNA stability of PSMD14. In order to identify the specific m5C modification sites of ALKBH1 for PSMD14, we predicted the m5C modification sites present in PSMD14 through Rnanut online database. Then we conducted a dual fluorescence assay by mutating sites 1# (site 147), 2# (site 370), and 3# (site 1326) pf PSMD14. The results showed that the fluorescence activity of sites 1# and sites 3# of PSMD14 were significantly changed after ALKBH1 knockdown, and the fluorescence activity of the PSMD14 gene mutated at sites 1# and sites 3# was not regulated by ALKBH1 expression. Interestingly, we found that the fluorescence activity of sites 1# of PSMD14 showed the most significant change after ALKBH1 knockdown. From this, it can be seen that ALKBH1 may modify PSMD14 with m5C at site 147, thereby regulating the expression of PSMD14. Finally, the rescue experiment demonstrated that PSMD14 knockdown reversed the function of ALKBH1 silencing in the hypoxia treated HTR-8/SVneo cells.

In conclusion, this study demonstrated that ALKBH1 knockdown promoted the cell growth, migration, and invasion of hypoxia treated HTR-8/SVneo cells through enhancing the m5C levels of PSMD14. ALKBH1 and PSMD14 might be promising targets for PE treatment. However, the absence of validation using publicly available patient datasets is a limitation of our current work. Moving forward, we are committed to addressing this limitation. We plan to seek out appropriate patient datasets or collaborate with clinical partners to collect relevant samples, which will allow us to validate our findings in a more clinically relevant context. This next phase of research will be crucial in translating our mechanistic insights into potential diagnostic or therapeutic strategies for PE.

## Electronic supplementary material

Below is the link to the electronic supplementary material.

Supplementary Material 1

## Data Availability

The datasets used and/or analysed during the current study are available from the corresponding author on reasonable request.
