# Supplementary material for: ALKBH1 knockdown promotes the growth, migration and invasion of HTR-8/SVneo cells through regulating the m5C modification PSMD14
Source: Sci Rep. 2025 Mar 1;15:7345. doi: 10.1038/s41598-025-91233-3 (PMC11873043; doi:10.1038/s41598-025-91233-3)
Supplement: Supplementary file 1 — Supplementary Material 1 [file 41598_2025_91233_MOESM1_ESM.docx]

Fig.1C

|  | Nor | Hyp |
| --- | --- | --- |
| ALKBH5 | 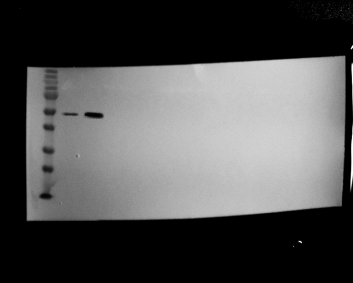 | |
| β-actin | 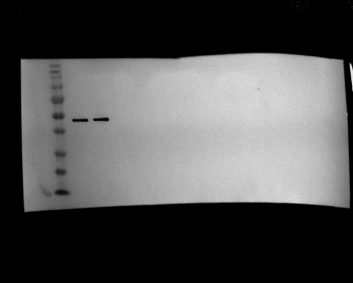 | |

Fig.3C

|  | Nor | Hyp | Hyp+shNC | Hyp+shALKBH1 |
| --- | --- | --- | --- | --- |
| PSMD14 | 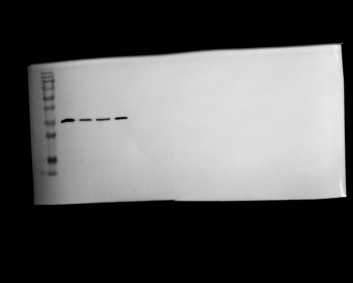 | | | |
| β-actin | 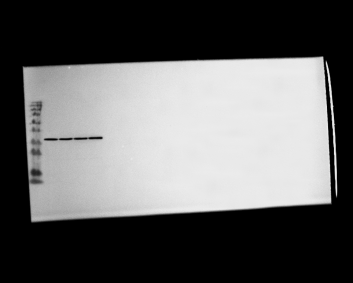 | | | |

Fig.4

|  | shNC | shPSMD14 |
| --- | --- | --- |
| PSMD14 | 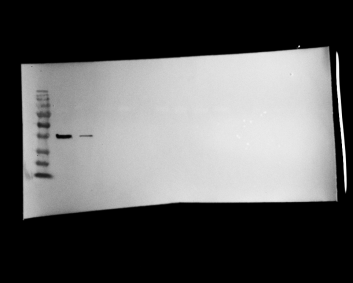 | |
| β-actin | 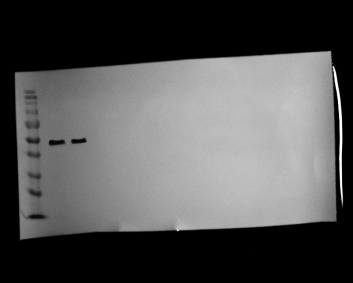 | |
